# Predicting Survival after Liver Transplantation Based on Pre-Transplant MELD Score: a Systematic Review of the Literature 

**DOI:** 10.1371/journal.pone.0080661

**Published:** 2013-12-12

**Authors:** Kristin B. Klein, Taenia D. Stafinski, Devidas Menon

**Affiliations:** 1 Faculty of Medicine & Dentistry, University of Alberta, Edmonton, Alberta, Canada; 2 School of Public Health, University of Alberta, Edmonton, Alberta, Canada; Karolinska Institutet, Sweden

## Abstract

The model for end-stage liver disease (MELD) score is used to stratify candidates for liver transplantation based on objective measures of disease severity. MELD has been validated as a predictor of wait-list mortality in transplantation candidates and has been postulated as a predictor of post-transplant survival. The purpose of this study was to examine the predictive value of the pre-transplantation MELD score on post-transplant survival from relevant existing studies. A systematic review and critical appraisal was performed using Cochrane guidelines. PubMed, the Cochrane Library, Embase, and Web of Science were searched for articles published in the English language since 2005 using a structured search strategy. There were 3058 discrete citations identified and screened for possible inclusion. Any study examining the relationship between pre-transplant MELD and post-transplant survival in the general transplant population was included. Thirty-seven studies met these criteria and were included in the review. Studies were all case series that typically involved stratified analyses of survival by MELD. They represented 15 countries and a total of 53,691 patients. There was significant clinical heterogeneity in patient populations across studies, which precluded performance of a meta-analysis. In 15 studies, no statistically significant association between MELD and post-transplant survival was found. In the remaining 22, some association was found. Eleven studies also measured predictive ability with c-statistics. Values were below 0.7 in all but two studies, suggesting poor predictive value. In summary, while the majority of studies reported an association between pre-transplantation MELD score and post-transplant survival, they represented a low level of evidence. Therefore, their findings should be interpreted conservatively.

## Introduction

The identification of patients who are most likely to benefit from orthotopic liver transplantation (OLT) is a significant challenge in transplantation medicine. Liver transplantation offers the only curative therapy for patients with end-stage liver disease (ESLD). However, the supply of donor livers remains inadequate to meet the demand, necessitating an effective policy for organ allocation. In order to minimize waitlist mortality, a Model for End-Stage Liver Disease (MELD) - based organ allocation was proposed. First adopted by the United States in February 2002, it has become one of the most widely used approaches to prioritizing liver transplant candidates in countries around the world. The MELD score was initially developed by Malinchoc et al [1] to predict mortality in patients undergoing transjugular intrahepatic portosystemic shunts, but has since been validated as predictor of short-term mortality in patients awaiting transplantation. It uses objective variables (creatinine, bilirubin and the international normalized ratio of prothrombin time (PTINR)) to quantify the severity of ESLD, enabling the prioritization of patients in need of liver transplantation by medical urgency. An ideal system would allocate organs to patients not only at the highest risk of dying without transplantation, but also with the highest likelihood of survival following transplantation. 

In recent years, the possibility of using pre-transplant MELD to predict post-transplant survival has been explored in many opinion pieces and expert reviews[2]. However, it has yet to be assessed through a systematic review and critical appraisal of published studies that adheres to internationally accepted systematic review guidelines. The need for such a review is heightened by the lack and infeasibility of RCTs on this topic and the fact that considerable debate over the value of MELD in this context remains. Thus, the aim of this study was to assess the association and predictive value of pre-transplantation MELD score on post-transplantation patient survival through a comprehensive, protocol driven systematic review of studies published to date. 

## Methods

### Identification of potentially relevant studies

To identify relevant studies published as of August 2011, a structured search strategy combining relevant controlled vocabulary terms such as Medical Subject Headings (MeSH) and additional non-indexed terms was first developed. Such terms included Model for End-Stage Liver Disease, MELD, liver transplantation, liver failure, and survival. The search strategy was applied to the following electronic bibliographic databases: PubMed (MEDLINE and non-MEDLINE), the Cochrane Library, EMBASE, and Web of Science; and limited to full text, English language studies of adult patients which were published within the past 10 years. For completeness, reference lists of relevant articles were scanned. Also, an internet search for unpublished studies was performed with the Google® search engine. Full search details are provided in Table A in File S1. 

### Selection of studies for inclusion in the review

Two researchers independently screened the titles and abstracts of citations identified through the literature search using predetermined inclusion criteria (Table 1). The initial search strategy identified studies published in the last ten years, whereas only studies published since 2005 were included in the review. 

**Table 1:** Inclusion/exclusion criteria for review.

**Parameter**	**Inclusion Criteria**	**Exclusion Criteria**
**General**	Full-text articles published in the English language since 2005	Abstracts
**Participants**	Adults patients with liver failure	Patient populations not representing the general liver transplant population (ex: patients with HCC or HCV only)
**Intervention**	Patient’s first orthotopic, whole liver, deceased-donor transplant	Multi-organ transplants, non-standard donor or living donor transplants, split liver transplants, sequential transplants
**Comparator**	Pre-operative Model for end-stage liver disease score	Delta-Meld, MELD-Na, post-operative MELD score
**Outcome**	Post-transplantation patient survival	Survival rate not reported by MELD score
**Study design**	Cohort, cross sectional, RCT, quasi-RCT, or controlled studies	Case study or series, commentaries, and opinion pieces without primary data

Since the purpose of this study was to assess the predictive ability of MELD in the general transplant patient population, studies focussing on specific subgroups of patients were excluded, along with those involving only unique transplant conditions, such as multi-organ transplants, split livers, and non-standard donors. However, studies that considered these conditions within the context of the broader transplantation population were included in the review. Corresponding papers of citations deemed potentially relevant were then retrieved for full review. The level of consensus among reviewers was assessed using the Kappa Statistic. A score of 0.98 was achieved, indicating excellent agreement. Discrepancies among reviewers were resolved through discussion without the need for third party adjudication. 

### Extraction of data from included studies

Information from included studies was systematically extracted using a pre-tested data abstraction form. The abstraction form contained elements related to study design, patient population, comparators, outcomes measured, and findings. All studies were reviewed by the primary author, with a second reviewer extracting information on 50% of the studies. Reviewers subsequently met to compare results. No discrepancies were found. Therefore, a second, independent review of the remaining studies was deemed unnecessary.

### Critical appraisal of included studies

Studies were appraised using the Oxford Center for Evidence-based Medicine Levels and Grades of Recommendation[3]. 

### Data analysis and synthesis of results

Extracted data were tabulated to facilitate a comparison of findings across studies. A meta-analysis of pre-transplant MELD on post-transplant survival using a random effects model was also planned (see Results section). Prior to presenting pooled or summary estimates, clinical heterogeneity and statistical heterogeneity using the I^2^ statistic were assessed. 

## Results

**Figure 1:**
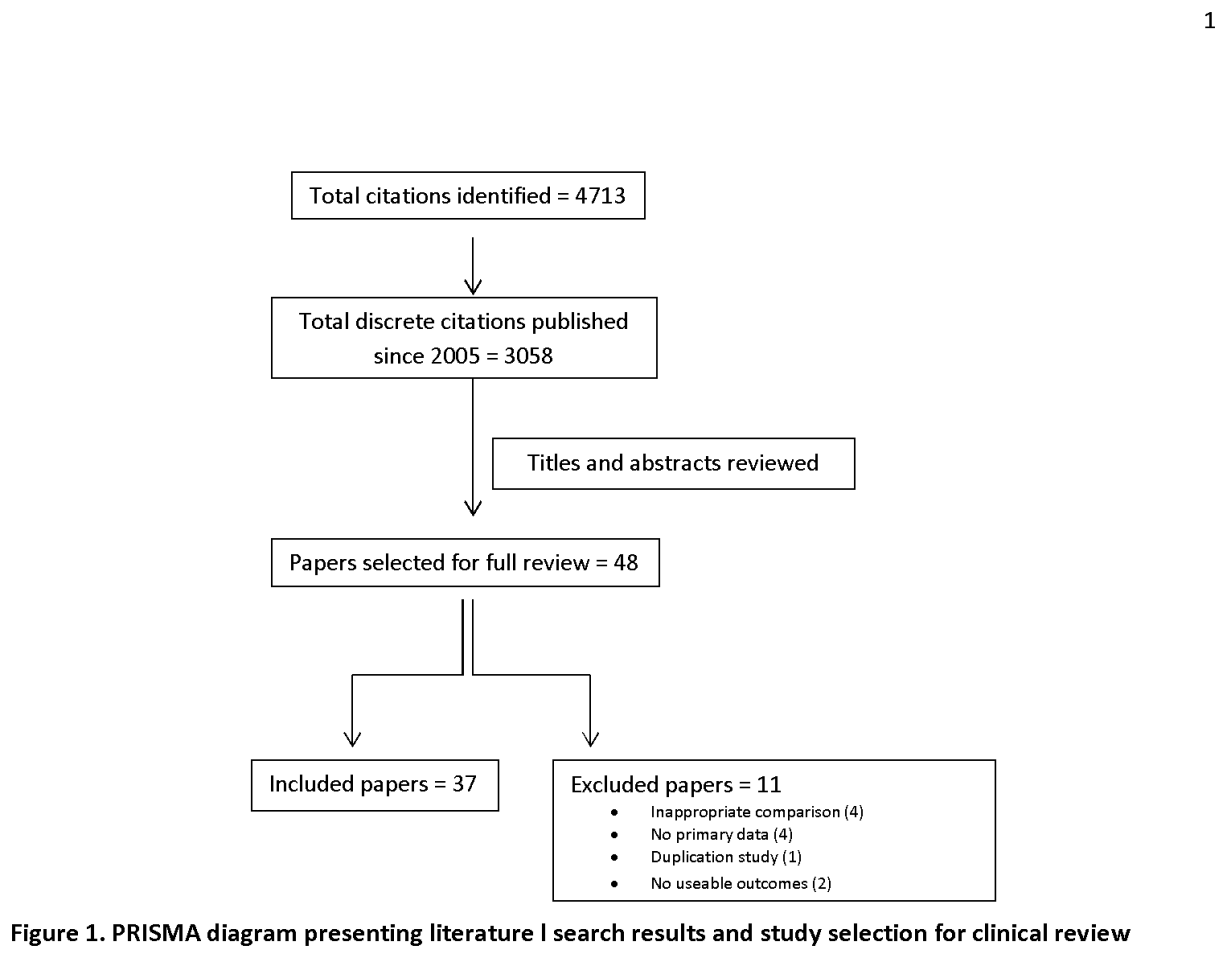
Literature search results and study selection for clinical review.

Results of the literature search are presented in the PRISMA diagram (Figure 1). The search yielded 3058 discrete citations. Forty-eight full-text articles were retrieved for full consideration, of which 37 met the inclusion/exclusion criteria of the review. Among excluded studies, 6 involved inappropriate comparators or outcomes, 4 did not present primary data, and 1 contained data already captured in an included study. The list of excluded studies, along with reasons for exclusion, is presented in Table B in File S1. 

### Description of included studies

The 37 studies comprised both prospective and retrospective case series, and collectively included a total of 53,691 patients. In most, the main objective was not to assess the relationship between MELD and post-transplant survival. Instead, studies examined a broad range of pre-transplant factors that may or may not influence survival through exploratory analyses. Studies originated from several countries, including: Belgium (2), New Zealand (1), the United Kingdom (3), Brazil (6), Spain (5), the United States (7), Singapore (1), Turkey (1), China (1), Korea (1), Switzerland (1), Poland (1), Italy (3), Canada (1), and Germany (3). The majority were single centered. Sample sizes ranged from 46 to 21,673 patients (mean = 1451, median = 222), most of whom were male. None used a power calculation to determine sample size. Sampling methods comprised consecutive patients who met inclusion/exclusion criteria, which differed considerably across studies. Therefore, patients comprising the “general transplant population” may have varied. The point at which MELD was measured in patients was inconsistent across studies, with some using time of placement on the transplant list and others using time of transplant. In most of the studies, the relationship between MELD and survival was examined through stratified analyses of survival across sub-groups defined by MELD score, where MELD cut-off points for sub-groups were determined post-hoc. Further, the majority(25) measured the association between MELD and survival based on univariate analyses alone, and, therefore, did not control for potential confounders. Eleven of the studies assessed the predictive ability of the MELD score on post-transplantation survival using a receiver operating characteristic (ROC) curve and the c-statistic. In 4 of the studies, there was partial overlap of patient populations since they included data from the Transplant Scientific Registry (Cywinski et al [4], Freeman et al [5], Rana et al [6] and Yoo & Thuluvath [7]). All of these studies were kept in the review as each used different MELD categories and follow-up times in their analysis. A detailed summary of each study is presented in Table C in File S1. 

As mentioned above, the majority of studies grouped patients by MELD. Specifically, MELD, which represents a continuous variable, was converted to a categorical variable for the analyses. The cut-off points for such categories varied widely across studies and were typically determined post-hoc. (Refer to relevant outcome measures in Table C in File S1). Effect measures also differed, ranging from proportions to hazards ratios, odds ratios, and relative risks, and follow-up time periods were inconsistent. Lastly, characteristics of the “general transplant population” varied. Therefore, given such clinical heterogeneity across studies, a meta-analysis was deemed inappropriate, and a statistical assessment of heterogeneity was not performed. 

### Quality of included studies

Based on the Oxford Center for Evidence-based Medicine Levels of Evidence, the quality of all of the included studies was level IV. The studies, which involved a comparison of pre-transplant MELD scores with post-transplant survival, were all case series and predominantly retrospective in design. All studies recruited consecutive patients over a specified time period, thereby reducing the risk of selection bias. 

### Association between Pre-Transplant MELD Score and Post-Transplantation Survival

Of the 37 studies, 15 found no association between pre-transplant MELD score and post-transplant patient survival, while 22 reported poorer survival with higher MELD. A detailed description of the results of each study is presented in Table C in File S1. Based on qualitative analyses, there were no clear differences in studies with statistically significant findings compared to those with no statistically significant findings. In both groups, sample sizes varied, as did follow-up times. However, findings from the two largest studies (N >15,000) both suggested that survival decreased with increasing MELD. One observed this relationship only when patients with MELD scores under 9 were compared to those with scores of 30 or greater, while the other had treated MELD as a continuous variable. At the same time, of the 7 other studies that analysed MELD as a continuous variable, all but one found no statistically significant association between MELD and survival. In most of the studies, information presented on patient characteristics was limited. Therefore, it was not possible to identify any differences in patient populations that could explain inconsistencies in the findings. 

### Predictive ability of MELD score for post-transplantation survival

Eleven studies presented a receiver operating characteristic (ROC) curve to determine the predictive ability of pre-transplant MELD score to determine post-transplantation survival. The area under the curve is used to produce a concordance value called the c-statistic. A c-statistic of 0.50 indicates no predictive ability, and is expected if the results are due to chance alone. In contrast, a c-statistic of 1 represents perfect discrimination. Values under 0.7 suggest poor predictive power, while those greater than 0.70 indicate a useful test, and those higher than 0.80 imply excellent predictive accuracy[2]. Among the 11 studies, 10 reported c-statistics less than <0.7, indicating that MELD poorly predicted post-transplant survival. This included the largest study contributing to the review[6]. In 1 study, the c-statistic decreased over time, from 0.711 for 3-month post-transplant survival to 0.679 for 12-month survival[8]. In the single study with a high c-statistic[9], there was no clear difference in sample size, follow-up time, or patient population when compared to the studies with lower values. 

## Discussion

This review assessed the association and predictive ability of pre-transplantation MELD score on post-transplantation survival in adults with end-stage liver disease. It highlighted discrepancies in findings across studies. Such discrepancies may be related to the nature of the studies, The vast majority were retrospective case series that relied upon exploratory stratified analyses of data to detect a relationship between MELD and post-transplant survival. As such, analytical techniques, rather than study design, were used to control for confounding. In addition, most of the studies were single centered, with each site having its own process for prioritizing patients for transplant. Therefore, patients constituting the general transplant population may have varied across studies. Thus, while using pre-transplant MELD to predict post-transplant survival in transplant candidates may be attractive, there is little evidence to support it. Prospective studies designed specifically to examine this relationship are needed. MELD score does appear to have a greater impact on mortality when observed in combination with other known risk factors for post-transplant mortality, including sub-optimal livers, low graft-to-body ratio and presence of Hepatitis C. Further research in the area of particular patient subgroups (such as those with hepatitis C) may show a stronger association between MELD and post-transplant outcome. 

Based on the studies conducted to date, which collectively represent a low level of evidence, MELD could be correlated with survival, but appears to have limited predictive ability. The vast majority of studies presenting concordance statistics found that pre-transplant MELD score offered minimal discriminating power for post-transplantation survival. However, the c-statistic may be of limited value in determining the predictive ability[10]. This is because the c-statistic is intended for diagnostic models, rather than prognostic models. The two types differ in that prognostic models add the element of time. Specifically, diagnostic models are designed to determine the current state of the patient and accurately identify an existing disease state. In contrast, prognostic models are designed to estimate the probability of a future state where the outcome is not yet known and subject to chance. 

This review is limited by heterogeneity in key parameters of studies used to date, which precluded performance of a meta-analysis. Studies reported different comparators (in terms of MELD categories) and applied time-points for outcomes. Studies comparing standardized MELD categories would be beneficial in determining whether or not there is, in fact, a threshold level at which liver transplantation does not offer sufficient survival to warrant the use of scarce donor livers. A more accurate assessment of post-transplant survival would also need to look at other factors, such as quality of life. Research examining the combinations of patient factors using more appropriate statistical techniques may also be valuable in improving the predictive ability of pre-transplant elements on post-transplant outcome. 

## Conclusions

This study provides a comprehensive review of recent articles examining the relationship between pre-operative MELDS score and post-transplantation survival. Based on the results of studies conducted to date, it appears that the use of MELD does not serve as a reliable predictor of post-transplantation survival. This may be a reflection of a reliance on less than ideal analytical measures. However, the use of pre-transplant characteristics may always fall short of ensuring optimal organ allocation due to variability in immeasurable patient factors and the complexity of perioperative and postoperative conditions. 

## Supporting Information

File S1 **Table A in File S1.** Literature Search. **Table B in File S1.** Excluded Studies. **Table C in File S1**. Description of Included Studies
(DOC)

Checklist S1 **Prisma checklist.**
(DOC)
