# Correction: Predicting Survival after Liver Transplantation Based on Pre-Transplant MELD Score: a Systematic Review of the Literature 

**DOI:** 10.1371/annotation/d01fbea0-579a-4ebd-bd03-e76df82b757e

**Published:** 2013-12-31

**Authors:** Kristin B. Klein, Tania D. Stafinski, Devidas Menon

The name of the second author is incorrect. The correct name is: Tania D. Stafinski.

