# Supplementary material for: Predicting Survival after Liver Transplantation Based on Pre-Transplant MELD Score: a Systematic Review of the Literature
Source: PLoS One. 2013 Dec 12;8(12):e80661. doi: 10.1371/journal.pone.0080661 (PMC3861188; doi:10.1371/journal.pone.0080661)
Supplement: File S1 — Table A in File S1. Literature Search. Table B in File S1. Excluded Studies. Table C in File S1. Description of Included Studies (DOC) [file pone.0080661.s001.doc]

**Table A. Literature search**

1. Pubmed (August 8, 2011)

| **Search** | | **Result** |
| --- | --- | --- |
| [#21](http://www.ncbi.nlm.nih.gov/pubmed/?querykey=21&dbase=pubmed&querytype=eSearch&) | Search #18 OR #19 OR #20 Limits: published in the last 10 years | 3539 |
| [#20](http://www.ncbi.nlm.nih.gov/pubmed/?querykey=20&dbase=pubmed&querytype=eSearch&) | Search #8 Limits: published in the last 10 years | 1610 |
| [#19](http://www.ncbi.nlm.nih.gov/pubmed/?querykey=19&dbase=pubmed&querytype=eSearch&) | Search #17 Limits: Humans, English, All Adult: 19+ years, published in the last 10 years | 2631 |
| [#18](http://www.ncbi.nlm.nih.gov/pubmed/?querykey=18&dbase=pubmed&querytype=eSearch&) | Search #17 AND (in process[sb] OR publisher[sb] OR pubmdenotmedline[sb]) | 57 |
| [#17](http://www.ncbi.nlm.nih.gov/pubmed/?querykey=17&dbase=pubmed&querytype=eSearch&) | Search #15 OR #16 | 7061 |
| [#16](http://www.ncbi.nlm.nih.gov/pubmed/?querykey=16&dbase=pubmed&querytype=eSearch&) | Search #5 AND #8 | 1094 |
| [#15](http://www.ncbi.nlm.nih.gov/pubmed/?querykey=15&dbase=pubmed&querytype=eSearch&) | Search #5 AND (#9 OR #10 OR #11 OR #12 OR #13 OR #14) | 6341 |
| [#14](http://www.ncbi.nlm.nih.gov/pubmed/?querykey=14&dbase=pubmed&querytype=eSearch&) | Search mortality[ti] AND “post-transplant*”[tiab] | 65 |
| [#13](http://www.ncbi.nlm.nih.gov/pubmed/?querykey=13&dbase=pubmed&querytype=eSearch&) | Search mortality[mh] | 234222 |
| [#12](http://www.ncbi.nlm.nih.gov/pubmed/?querykey=12&dbase=pubmed&querytype=eSearch&) | Search prognostic[ti] | 43582 |
| [#11](http://www.ncbi.nlm.nih.gov/pubmed/?querykey=11&dbase=pubmed&querytype=eSearch&) | Search survival[ti] | 78062 |
| [#10](http://www.ncbi.nlm.nih.gov/pubmed/?querykey=10&dbase=pubmed&querytype=eSearch&) | Search survival rate[mh] | 102733 |
| [#9](http://www.ncbi.nlm.nih.gov/pubmed/?querykey=9&dbase=pubmed&querytype=eSearch&) | Search outcome*[ti] | 130809 |
| [#8](http://www.ncbi.nlm.nih.gov/pubmed/?querykey=8&dbase=pubmed&querytype=eSearch&) | Search #6 OR #7 | 1650 |
| [#7](http://www.ncbi.nlm.nih.gov/pubmed/?querykey=7&dbase=pubmed&querytype=eSearch&) | Search MELD[tiab] | 1364 |
| [#6](http://www.ncbi.nlm.nih.gov/pubmed/?querykey=6&dbase=pubmed&querytype=eSearch&) | Search “model for end stage liver disease” | 1083 |
| [#5](http://www.ncbi.nlm.nih.gov/pubmed/?querykey=5&dbase=pubmed&querytype=eSearch&) | Search #1 OR #2 OR #3 OR #4 | 49990 |
| [#4](http://www.ncbi.nlm.nih.gov/pubmed/?querykey=4&dbase=pubmed&querytype=eSearch&) | Search “liver transplant*”[ti] | 3894 |
| [#3](http://www.ncbi.nlm.nih.gov/pubmed/?querykey=3&dbase=pubmed&querytype=eSearch&) | Search “liver failure*”[ti] | 2735 |
| [#2](http://www.ncbi.nlm.nih.gov/pubmed/?querykey=2&dbase=pubmed&querytype=eSearch&) | Search liver transplantation[mh] | 37254 |
| [#1](http://www.ncbi.nlm.nih.gov/pubmed/?querykey=1&dbase=pubmed&querytype=eSearch&) | Search liver failure[mh] | 15426 |

2. Embase (August 8, 2011)

| **Search** | | **Result** |
| --- | --- | --- |
| #1 | exp liver transplantation/ | 54865 |
| #2 | exp liver failure/ | 32482 |
| #3 | model for end-stage liver disease.mp. | 1407 |
| #4 | meld.mp. | 2834 |
| #5 | exp survival rate/ | 104347 |
| #6 | exp survival/ | 394422 |
| #7 | exp survival time/ | 44572 |
| #8 | exp prognosis/ | 347412 |
| #9 | exp treatment outcome/ | 740670 |
| #10 | exp mortality/ or exp surgical mortality/ | 470424 |
| #11 | 1 or 2 | 78740 |
| #12 | 3 or 4 | 3184 |
| #13 | 5 or 6 or 7 or 8 or 9 or 10 | 1589161 |
| #14 | 11 and 12 and 13 | 1822 |
| #15 | limit 14 to (human and 2nglish language and yr=”2000 –Current” and (adult <18 to 64 years> or aged <65+ years>)) | 678 |

3. The Cochrane Library (August 8, 2011)

| **Search** | | **Result** |
| --- | --- | --- |
| #1 | (liver transplantation):ti,ab,kw or (liver failure):ti,ab,kw | 3281 |
| #2 | (model for end-stage liver disease) or (MELD) | 217 |
| #3 | (#1 AND #2) | 69 |

4. Web of Science (August 8, 2011)

| **Search** | | **Result** |
| --- | --- | --- |
| #3 | #2 AND #1  Databases=SCI-EXPANDED, SSCI, A&HCI, CPCI-S, CPCI-SSH Timespan=2000-2011 | 427 |
| #2 | Title=(“liver transplant*”)  Databases=SCI-EXPANDED, SSCI, A&HCI, CPCI-S, CPCI-SSH Timespan=2000-2011 | 22,698 |
| #1 | Title=(MELD) OR Title=(“model for end-stage”)  Databases=SCI-EXPANDED, SSCI, A&HCI, CPCI-S, CPCI-SSH Timespan=2000-2011 | 1,201 |

Table B. Excluded studies

| **Study** | **Reason for exclusion** |
| --- | --- |
| Anderson et al (2008)[1] | Inappropriate comparison: associated post-transplant survival with donor age stratified by MELD |
| Brinceno et al (2008)[2] | Post-operative MELD scores instead of pre-operative MELD scores were used |
| Cholongitas et al (2006)[3] | Review article with no primary data |
| Foxton et al (2010)[4] | Duplicate patient population to Foxton et al (2006)(5) |
| Huo et al (2005)[5] | Reports pre-transplant instead of post-transplant survival |
| Maluf et al (2006)[6] | Reports graft failure, not patient survival |
| Martin et al (2007)[7] | Review article with no primary data |
| Merion et al (2005)[8] | Inappropriate comparison: compared survival with and without transplant by MELD category |
| Nadalin et al (2009)[9] | Inappropriate comparison: compared survival with split liver compared to whole liver transplant stratified by MELD |
| Perkins JD (2009)[10] | Commentary on an abstract |
| Perkins et al (2009)[11] | Decision modelling study design |

**Table C. Description of included studies**

|  | | | | | | | | | | | | | |
| --- | --- | --- | --- | --- | --- | --- | --- | --- | --- | --- | --- | --- | --- |
| **Study** | **Study Design** | **No. of patients** | **MELD** | **Survival (months)** | | | | | | | | | |
|  |  |  |  | 1 | 3 | 6 | 9 | 12 | 24 | 36 | 48 | 60 | Other |
| Adler et al (2005)[12] | Retrospective  case series | 137 | ≤17 *vs*  >17 | NR* | NR | No statistically significant difference  (p>0.05) | NR | NR | NR | NR | NR | No statistically significant difference  (p>0.05) |  |
| Al-Freah et al (20110[13] | Retrospective case series | 226 | <15 *vs*  15-20 *or*  >20 | NR | No statistically significant difference (p=0.336) | NR | NR | No statistically significant difference (p=0.228) | NR | NR | NR | NR |  |
| Basile-Filho et al (2011)[14] | Retrospective case series | 63 | NR | C-statistic=0.50 indicating poor predictive value | NR | NR | NR | NR | NR | NR | NR | NR |  |
| Benckert et al (2011)[15] | Retrospective case series | 154 | 6-19 *vs*  20-29 *or*  ≥30 | NR | NR | NR | NR | No statistically significant difference (p=0.82) | NR | NR | NR | NR |  |
| Bonney et al (2009)[16] | Retrospective case series | 1090 | <15 *vs*  15-30 *or*  >30 | Higher MELD associated with poorer survival  (p < 0.05) | NR | NR | NR | Higher MELD associated with poorer survival  (p < 0.05) | NR | Higher MELD associated with poorer survival  (p < 0.05) | NR | 71.8%  NR  51.4%  Higher MELD associated with poorer survival  (p < 0.05)  (p < 0.05) |  |
| Brandao et al (2009)[17] | Retrospective case series | 436 | <21 *vs*  ≥21 | NR | HR† = 2.2  (p < 0.05)  Higher MELD associated with poorer survival  C-statistic 0.60 indicating poor predictive value | HR = 2.2  (p < 0.05)  Higher MELD associated with poorer survival | HR = 2.1  (p < 0.05)  Higher MELD associated with poorer survival | HR = 1.3 (p < 0.05)  Higher MELD associated with poorer survival |  |  |  |  |  |
| Buenadicha et al (2005)[18] | Retrospective case series | 168 | <24 *vs*  >24 | No statistically significant difference  (p>0.05) | No statistically significant difference  (p>0.05) |  |  |  |  |  |  |  |  |
| Cywinski et al (2011)[19] | Retrospective case series | 15156 | <18 *vs*  18-30 *or*  >30 | HR = 1.44  (for MELD as a continuous variable) | NR | NR | NR | NR | NR | HR = 1.56  HR = 1.14 | NR | NR |  |
| Emiroglu et al (2007)[20] | Retrospective case series | 62 | <20 *vs*  ≥20 | NR | NR | NR | NR | NR | NR | NR | NR | NR | •MELD score associated with early mortality •Highest mortality (44%) associated with MELD ≥20 & low graft-to-body-weight ratio |
| Ferraz-Neto (2008)[21] | Retrospective case series | 111 | <30 *vs*  ≥30 | No statistically significant difference (p = 0.688) | NR | NR | NR | NR | NR | NR | NR | NR |  |
| Foxton et al (2006)[22] | Retrospective case series | 402 | NR | NR | NR | NR | NR | NR | NR | NR | NR | NR | No statistically significant difference in overall survival by MELD score (RR 1.082, p=1.182) |
| Freeman et al (2005)[23] | Retrospective case series | 3499 | 7-15  16-25  26-35  >35 | NR | NR | NR | NR | 89.5%  88.3%  86.6%  78.1%  (p = 0.0002)  Higher MELD associated with poorer survival | NR | NR | NR | NR |  |
| Gleisner et al (2010)[24] | Prospective case series | 520 | Continuous variable | NR | NR | NR | NR | NR | NR | NR | NR | NR | • Median survival decreased by 12% with each unit increase in MELD (95% CI: -20% to -2%) |
| Guo et al (2010)[25] | Retrospective case series | 117 | NR | NR | Higher MELD associated with poorer survival  (p < 0.05)  C-statistic = 0.950 indicating excellent predictive value | NR | NR | Higher MELD associated with poorer survival  (p < 0.05)  C-statistic = 0.868 indicating excellent predictive value | NR | NR | NR | NR |  |
| Habib et al (2006)[26] | Retrospective case series | 1472 | 6-15 *vs*  16-25 *or*  >25 | NR | NR | NR | NR | RR‡ = 0.52  RR = 0.53 | NR | NR | NR | NR | • C-statistic for overall survival = 0.63 indicating poor predictive value |
| Kim et al (2006)[27] | Retrospective case series | 98 | Continuous variable | NR | NR | NR | NR | NR | NR | NR | NR | NR | • No statistically significant difference in overall survival (p>0.05) |
| Lee et al (2006)[28] |  | 46 | NR | NR | NR | Statistically significant difference (p=0.037) | NR | No statistically significant difference (p = 0.065) | NR | NR | NR | NR |  |
| Manduca Palmiero et al (2010)[29] | Retrospective case series | 1786 | <25 *vs*  ≥25 | NR | NR | NR | NR | NR | NR | NR | NR | NR | • Higher MELD associated with poorer overall survival  (HR 1.029, p=0.0001) |
| Nagler et al (2005)[30] | Retrospective case series | 121 | <30 *vs*  ≥30 | NR | NR | NR | NR | Higher MELD associated with poorer survival  (p=0.02) | NR | NR | NR | NR | • C-statistic for 1- 12 year mortality ≤0.61 indicating poor predictive value |
| Oberkofler et al (2010)[31] | Retrospective case series | 144 | Continuous variable | NR | NR | NR | NR | NR | NR | NR | NR | NR | • No statistically significant difference in overall survival  (p=0.26) |
| Onaca et al (2006)[32] | Retrospective case series | 669 | 6-14  15-24  25-40 | NR | NR | NR | NR | NR | NR | NR | 85.9%  82.3%  74.8%  (p<0.001)  Higher MELD associated with poorer survival | NR |  |
| Patkowski et al (2009)[33] | Retrospective case series | 215 | <20 *vs*  ≥20 | NR | NR | NR | NR | Higher MELD associated with poorer survival  (p=0.0001) |  | Higher MELD associated with poorer survival    (p=0.0001) |  | Higher MELD associated with poorer survival  (p=0.0001) |  |
| Rana et al (2008)[34] | Retrospective case series | 21673 | <9 *vs*  30-39 *or*  ≥40 | NR | OR§ = 1.4 (p<0.001)  OR = 1.4  (p=0.007)  Higher MELD associated with poorer survival  C-statistic = 0.63 indicating poor predictive value | NR | NR | NR | NR | NR | NR | NR |  |
| Ravaioli et al (2009)[35] | Prospective case series | 218 | <20 *vs*  ≥20 | NR | NR | NR | NR | NR | NR | Higher MELD associated with poorer survival  (p<0.05) | NR | NR |  |
| Sanchez-Perez et al (2005)[36] | Retrospective case series | 250 | ≤18 *vs*  >18 | NR | NR | NR | NR | Higher MELD associated with poorer survival  (P<0.01) | NR | NR | NR | NR |  |
| Santori et al (2005)[37] | Prospective case series | 69 | Continuous variable | No statistically significant difference (p=0.779)  C-statistic = 0.577 indicating poor predictive value | No statistically significant difference  (p=0.57)  C-statistic = 0.637 indicating poor predictive value | C-statistic = 0.57 indicating poor predictive value | NR | C-statistic = 0.543 indicating poor predictive value | NR | NR | NR | NR |  |
| Santoyo et al (2006)[38] | Retrospective case series | 197 | <10 *vs*  ≥10 | NR | NR | NR | NR | NR | NR | NR | NR | NR | • No statistically significant difference in overall survival (p>0.05) except at 8-years(MELD <10: 93% vs ≥10: 74%; p<0.01) |
| Silberhumer et al (2006)[39] | Retrospective case series | 300 | Continuous variable | NR | NR | NR | NR | NR | NR | NR | No statistically significant difference  (p=0.07) | NR |  |
| Siniscalchi et al (2009)[40] | Retrospective case series | 242 | <14 *vs*  ≥14 | NR | NR | NR | NR | NR | NR | NR | NR | NR | Higher MELD associated with poorer overall survival  (p<0.05) |
| Vitale et al (2007)[41] | Retrospective case series | 160 | ≤17 *vs*  >17 | NR | NR | NR | NR | NR | NR | NR | NR | NR | • Patients receiving sub-optimal liver: Higher MELD associated with poorer overall survival  (p=0.01)  • Patients receiving optimal liver: no statistically significant difference |
| Vrochides et al (2011)[42] | Retrospective case series | 458 | NR | NR | OR 1.054 p<0.001)  C-statistic = 0.614 indicating poor predictive value | NR | NR | NR | NR | NR | NR | R | • No statistically significant difference in long term survival (p=0.44). |
| Washburn et al (2006)[43] | Retrospective case series | 222 | Continuous variable | NR | NR | NR | NR | No statistically significant difference  (p=0.57) | NR | NR | NR | NR | • No statistically significant difference in overall survival  (p=0.57) |
| Weismuller et al (2008)[44]^a^ | Retrospective case series | 133 | ≤16  >16 | NR | NR | No statistically significant difference  (p=0.076) |  | 86.2%  71.7%  (p=0.041)  Higher MELD associated with poorer survival | NR | NR | NR | NR | • No statistically significant difference in overall survival (p=0.087) |
| Weismuller et al (2011)[45] ^a^ | Retrospective case series | 462 | ≤30 *vs*  >30 | NR | C-statistic= 0.711 indicating reasonable predictive value | NR | NR | OR: 4.17, CI: 2.57-6.78  Higher MELD associated with poorer survival  C-statistic = 0.679 indicating poor predictive value | NR | NR | NR | NR |  |
| Wiederkehr et al (2010)[46] | Retrospective case series | 155 | Continuous variable | NR | NR | NR | NR | Statistically significant difference  (p=0.046) C-statistic = 0.610 | NR | NR | NR | NR |  |
| Yoo & Thuluvath (2005)[47] | Retrospective case series | 2038 | <30 *vs*  ≥30 | NR | NR | NR | NR | NR | NR | NR | NR | NR | • 10 months survival: HR: 2.90, CI 1.50-5.61  Higher MELD associated with poorer survival |
| Young et al (2007)[48] | Retrospective case series | 422 | Continuous variable | NR | NR | NR | NR | Higher MELD associated with poorer survival  (p<0.05) | NR | Higher MELD associated with poorer survival  (p<0.05) | NR | NR | • C-statistic for overall survival = 0.58 indicating poor predictive value |
| *Not reported  †Hazard ratio  ‡Relative risk  §Odds ratio | | | | | | | | | | | | | |

^a^ some overlap in patient populations between Weismuller et al (2008)[44] & Weismuller et al (2010)[45]

**Reference List**

1. Anderson CD, Vachharajani N, Doyle M, Lowell JA, Wellen JR, et al. (2008) Advanced Donor Age Alone Does Not Affect Patient or Graft Survival after Liver Transplantation. Journal of the American College of Surgeons 207(6):847-52
2. Briceno J, Sanchez-Hidalgo JM, Naranjo A, Ciria R, Pozo JC, et al. (2008) Model for End-stage Liver Disease Can Predict Very Early Outcome After Liver Transplantation. Transplantation Proceedings 40(9):2952-4
3. Cholongitas E, Marelli L, Shusang V, Senzolo M, Rolles K, et al. (2006) A systematic review of the performance of the model for end-stage liver disease (MELD) in the setting of liver transplantation. Liver Transpl 12(7):1049-61
4. Foxton MR, Al-Freah MAB, Portal AJ, Sizer E, Bernal W, et al. (2010) Increased model for end-stage liver disease score at the time of liver transplant results in prolonged hospitalization and overall intensive care unit costs. Liver Transplantation 16(5):668-77.
5. Huo TI, Lin HC, Wu JC, Lee FY, Hou MC, et al. (2005) Different model for end-stage liver disease score block distributions may have a variable ability for outcome prediction. Transplantation 80(10):1414-8.
6. Maluf DG, Edwards EB, Kauffman HM. (2006) Utilization of extended donor criteria liver allograft: Is the elevated risk of failure independent of the Model for End-Stage Liver Disease score of the recipient? Transplantation 82(12):1653-7
7. Martin AP, Bartels M, Hauss J, Fangmann J.(2007)Overview of the MELD score and the UNOS adult liver allocation system. Transplant Proc 39(10):3169-74.
8. Merion RM, Schaubel DE, Dykstra DM, Freeman RB, Port FK, Wolfe RA. (2005) The survival benefit of liver transplantation. American Journal of Transplantation 5(2):307-13
9. Nadalin S, Schaffer R, Fruehauf N. (2009) Split-liver transplantation in the high-MELD adult patient: are we being too cautious? Transpl Int 22(7):702-6.
10. Perkins JD. (2009) Use of High-Risk Liver Allografts Lowers Survival in Patients with Low Model for End-Stage Liver Disease Scores Impact of the Model for End-Stage Liver Disease allocation policy on the use of high-risk organs for liver transplantation. Liver Transplantation 15(2):258-9.
11. Perkins JD.(2009) Use of High-Risk Liver Allografts Lowers Survival in Patients with Low Model for End-Stage Liver Disease Scores Impact of the Model for End-Stage Liver Disease allocation policy on the use of high-risk organs for liver transplantation. Liver Transplantation 15(2):258-9
12. Adler M, De GE, Vereerstraeten P, Degre D, Bourgeois N, et al. (2005) Value of the MELD score for the assessment of pre- and post-liver transplantation survival. Transplant Proc 37(6):2863-4
13. Al-Freah MA, Gane EJ, Livingstone V, McCall J, Munn S. (2011) The effect of changes of model for end-stage liver disease score during waiting time on post-liver transplant mortality. Hepatol Int Jun 30.
14. Basile-Filho A, Nicolini EA, Auxiliadora-Martins M, Alkmim-Teixeira GC, Martinez EZ, et al. (2011) Comparison of acute physiology and chronic health evaluation II death risk, Child-Pugh, Charlson, and Model for End-stage Liver Disease indexes to predict early mortality after liver transplantation. Transplantation Proceedings 43(5):1660-4.
15. Benckert C, Quante M, Thelen A, Bartels M, Laudi S, et al. (2011) Impact of the MELD allocation after its implementation in liver transplantation. Scandinavian Journal of Gastroenterology 46(7-8):941-8.
16. Bonney GK, Aldersley MA, Asthana S, Toogood GJ, Pollard SG, et al. (2009) Donor risk index and MELD interactions in predicting long-term graft survival: A single-centre experience. Transplantation 87(12):1858-63.
17. Brandao A, Fuchs SL, Gleisner AL, Marroni C, Zanotelli ML, et al. (2009) MELD and other predictors of survival after liver transplantation. Clinical Transplantation 23(2):220-7.
18. Buenadicha AL, Martin LG, Martin EE, Pajares ADP, Perez AM, et al.(2005) Assessment of short-term survival after liver transplant by the model for end-stage liver disease. Transplantation Proceedings 37(9):3881-3.
19. Cywinski JB, Mascha EJ, You J, Sessler DI, Kapural L, et al. (2011) Pre-transplant MELD and sodium MELD scores are poor predictors of graft failure and mortality after liver transplantation. Hepatol Int Feb 17.
20. Emiroglu R, Yilmaz U, Coskun M, Karakayali H, Haberal M. (2007) Higher Graft-to-Host Ratio May Decrease Posttransplant Mortality in Patients With a High MELD Score. Transplantation Proceedings 39(4):1164-5.
21. Ferraz-Neto BH, Zurstrassen MPVC, Hidalgo R, Meira-Filho SP, Rezende MB, et al. (2008) Analysis of Liver Transplantation Outcome in Patients With MELD Score >=30. Transplantation Proceedings 40(3):797-9.
22. Foxton MR, Kendrick S, Sizer E, Muiesan P, Rela M, et al. (2006) Change in model for end-stage liver disease score on the transplant waiting list predicts survival in patients undergoing liver transplantation. Transplant International 19(12):988-94.
23. Freeman RB, Harper A, Edwards EB. (2005) Excellent liver transplant survival rates under the MELD/PELD system. Transplant Proc 37(2):585-8.
24. Gleisner AL, Munoz A, Brandao A, Marroni C, Zanotelli ML, et al. (2010) Survival benefit of liver transplantation and the effect of underlying liver disease. Surgery 147(3):392-404.
25. Guo Z, He X, Wu L, Ju W, Hu A, et al. (2010) Model for end-stage liver disease versus the Child-Pugh score in predicting the post-transplant 3-month and 1-year mortality in a cohort of Chinese recipients. Surgery Today 40(1):38-45.
26. Habib S, Berk B, Chang C-C, Demetris AJ, Fontes P, et al. (2006) MELD and prediction of post-liver transplantation survival. Liver Transplantation 12(3):440-7.
27. Kim DJ, Lee S-K, Jo JW, Kim SJ, Kwon CHD, et al. (2006) Prognosis After Liver Transplantation Predicted by Preoperative MELD Score. Transplantation Proceedings 38(7):2095-6.
28. Lee YM, Fernandez M, Da CM, Lee KH, Sutedja DS, et al. (2006) MELD may not be the better system for organ allocation in liver transplantation patients in Singapore. Singapore Medical Journal 47(7):592-4.
29. Manduca Palmiero HO, Kajikawa P, Boin IFSF, Coria S, Pereira LA. (2010) Liver recipient survival rate before and after model for end-stage liver disease implementation and use of donor risk index. Transplantation Proceedings 42(10):4113-5.
30. Nagler E, Van VH, Colle I, Troisi R, de HB. (2005) Impact of MELD on short-term and long-term outcome following liver transplantation: A European perspective. European Journal of Gastroenterology and Hepatology 17(8):849-56.
31. Oberkofler CE, Dutkowski P, Stocker R, Schuepbach RA, Stover JF, et al. (2010) Model of end stage liver disease (MELD) score greater than 23 predicts length of stay in the ICU but not mortality in liver transplant recipients. Crit Care 14(3):R117
32. Onaca N, Levy MF, Ueno T, Martin AP, Sanchez EQ, et al. (2006) An outcome comparison between primary liver transplantation and retransplantation based on the pretransplant MELD score. Transplant International 19(4):282-7
33. Patkowski W, Zieniewicz K, Skalski M, Krawczyk M. (2009) Correlation Between Selected Prognostic Factors and Postoperative Course in Liver Transplant Recipients. Transplantation Proceedings 41(8):3091-102.
34. Rana A, Hardy MA, Halazun KJ, Woodland DC, Ratner LE, et al. (2008) Survival Outcomes Following Liver Transplantation (SOFT) score: A novel method to predict patient survival following liver transplantation. American Journal of Transplantation 8(12):2537-46.
35. Ravaioli M, Grazi GL, Dazzi A, Bertuzzo V, Ercolani G, et al. (2009) Survival benefit after liver transplantation: A single european center experience. Transplantation 88(6):826-34.
36. Sanchez-Perez B, Santoyo J, Fernandez-Aguilar JL, Suarez MA, Perez JA, et al. (2005) Preoperative factors and models predicting mortality in liver transplantation. Transplantation Proceedings 37(3):1499-501
37. Santori G, Andorno E, Morelli N, Antonucci A, Bottino G, et al. (2005) MELD score versus conventional UNOS status in predicting short-term mortality after liver transplantation. Transplant International 18(1):65-72.
38. Santoyo J, Suarez MA, Fernandez-Aguilar JL, Perez Daga JA, Sanchez-Perez B, et al. (2006) True Impact of the Indication of Cirrhosis and the MELD on the Results of Liver Transplantation. Transplantation Proceedings 38(8):2462-4
39. Silberhumer GR, Hetz H, Rasoul-Rockenschaub S, Peck-Radosavljevic M, Soliman T, et al. (2006) Is MELD score sufficient to predict not only death on waiting list, but also post-transplant survival? Transplant International 19(4):275-81.
40. Siniscalchi A, Cucchetti A, Toccaceli L, Spiritoso R, Tommasoni E, et al. (2009) Pretransplant Model for End-Stage Liver Disease Score as a Predictor of Postoperative Complications After Liver Transplantation. Transplantation Proceedings 41(4):1240-2.
41. Vitale A, D'Amico F, Brolese A, Zanus G, Boccagni P, et al. (2007) Prognostic Impact of Model for End-Stage Liver Disease Score in Patients Undergoing Liver Transplantation With Suboptimal Livers. Transplantation Proceedings 39(6):1907-9.
42. Vrochides D, Hassanain M, Barkun J, Tchervenkov J, Paraskevas S, et al. (2011) Association of preoperative parameters with postoperative mortality and long-term survival after liver transplantation. Canadian Journal of Surgery 54(2):101-6.
43. Washburn WK, Pollock BH, Nichols L, Speeg KV, Halff G. (2006) Impact of recipient MELD score on resource utilization. American Journal of Transplantation 6(10):2449-54.
44. Weismuller TJ, Prokein J, Becker T, Barg-Hock H, Klempnauer J, et al. (2008) Prediction of survival after liver transplantation by pre-transplant parameters. Scandinavian Journal of Gastroenterology 43(6):736-46
45. Weismuller TJ, Fikatas P, Schmidt J, Barreiros AP, Otto G, et al. (2011) Multicentric evaluation of model for end-stage liver disease-based allocation and survival after liver transplantation in Germany--limitations of the 'sickest first'-concept. Transpl Int 24(1):91-9.
46. Wiederkehr JC, Igreja MR, Nogara MS, Goncalves NL, Wiederkehr BA, et al. (2010) Analysis of Survival After Primary Liver Transplantation: Multivariate Analysis of 155 Cases in a Single Center. Transplantation Proceedings 42(2):511-2.
47. Yoo HY, Thuluvath PJ. (2005) Short-term postliver transplant survival after the introduction of MELD scores for organ allocation in the United States. Liver International 25(3):536-41.
48. Young AL, Rajagenashan R, Asthana S, Peters CJ, Toogood GJ, et al. (2007) The value of MELD and sodium in assessing potential liver transplant recipients in the United Kingdom. Transplant International 20(4):331-7.
